# The red blood cell as a novel regulator of human B‐cell activation

**DOI:** 10.1111/imm.13327

**Published:** 2021-05-06

**Authors:** Charlotte S. Lennon, Huan Cao, Andrew M. Hall, Mark A. Vickers, Robert N. Barker

**Affiliations:** ^1^ Institute of Medical Sciences University of Aberdeen Aberdeen UK

**Keywords:** B cell, CD22, human, immune regulation, red blood cell, sialic acid

## Abstract

Non‐immune cells are increasingly recognized as important in regulating immunity, but the role of red blood cells (RBC) remains relatively unexplored, despite their abundance in the circulation and a cell surface rich in potential ligands. Here, we determine whether RBC influence the activation state of human B cells. Separation of RBC from peripheral blood mononuclear cells increased B‐cell expression of HLA‐DR/DP/DQ, whilst reconstitution reduced the levels of B‐cell activation markers HLA‐DR/DP/DQ, CD86, CD69 and CD40, as well as decreasing proliferative responses and IgM secretion. Inhibition of B cells required contact with RBC and was abrogated by either removal of sialic acids from RBC or blocking the corresponding lectin receptor CD22 on B cells. Chronic lymphocytic leukaemia B cells express low levels of CD22 and were less susceptible to inhibition by RBC, which may contribute to their activated phenotype. Taken together, the results identify a novel mechanism that may suppress inappropriate responsiveness of healthy B cells whilst circulating in the bloodstream.

## Graphical abstract

Contact with red blood cells (RBC) suppresses the activation of human B‐cells. Separation of RBC from peripheral blood mononuclear cells increases B‐cell expression of HLA‐DR/DP/DQ, whilst reconstitution reduces the levels of B‐cell activation markers HLA‐DR/DP/DQ, CD86, CD69 and CD40, as well as decreasing proliferative responses and IgM secretion. Inhibition is mediated via ligation of the B‐cell lectin receptor CD22 by sialic acid on RBC, and represents a novel mechanism that may suppress inappropriate responsiveness of B‐cells while they are circulating in the bloodstream.

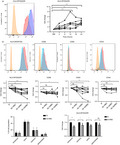

**Abbreviations**
APC: antigen‐presenting cell
CFSE: carboxyfluorescein succinimidyl ester
CLL: chronic lymphocytic leukaemia
Con A: concanavalin A
cpm: counts per minute
HEPES: *N*‐2‐hydroxyethylpiperazine‐*N*′‐2‐ethanesulphonic acid
KLH: keyhole limpet haemocyanin
PBMC: peripheral blood mononuclear cell
PPD: purified protein derivative
RBC: red blood cell
SI: stimulation indices
Siglec: sialic acid‐binding immunoglobulin‐like lectin
SLO: secondary lymphoid organ
αMEM: alpha modification of Eagle's medium

## INTRODUCTION

The importance for health of regulating adaptive immune responses is well established, particularly the roles of regulatory lymphocyte populations [1], but the contribution of non‐immune cells to this control is less well defined. For example, although the primary role of red blood cells (RBC) is gaseous transport, it is an open possibility that they have other functions, in particular the ability to modulate the activity of immune cells within their microenvironment [2]. RBC are by far the most abundant cell type in the bloodstream [3], where they are in continuous contact with circulating leucocytes and thus have the opportunity to deliver regulatory signals. In contrast to the extensive literature describing the immune effects of platelets [4], there are very few reports of the interactions of RBC with innate or adaptive immunity. Evidence has emerged that RBC can indeed inhibit immune cells such as neutrophils [5] and T cells [6], but little is known of their effects on human B cells.

B cells make up approximately 15% of circulating lymphocytes, and in addition to providing antibody responses, they function as professional antigen‐presenting cells (APCs), capable of activating antigen‐specific T cells [7]. It is an important question as to how B cells are regulated to ensure efficient and effective protection against pathogens, whilst constraining inappropriate activation that can lead to dysfunctional responses or autoimmune disease [8]. Uncontrolled proliferation and an activated phenotype are also hallmarks of B‐cell malignancies such as chronic lymphocytic leukaemia (CLL) [9].

Activation, proliferation and differentiation of adaptive immune cells are normally limited to secondary lymphoid organs (SLOs), such as the spleen and lymph nodes. The architecture of SLO is highly organized to optimize the interactions between B cells, T cells, APC and cognate antigen, providing an appropriate environment for the initiation and control of immune responses. Naïve B cells migrate through SLO for up to 24 h, and, if not activated, then return to the circulation to be taken back to SLO [10]. Activation by antigen in SLO and interactions with specific helper T cells lead to germinal centre formation, where B cells with high‐affinity antibodies can differentiate into plasma and memory cells [11].

Given the importance of SLO to the tight co‐ordination of efficient, appropriate adaptive immune responses, it seems likely there are mechanisms to constrain B‐cell activation in peripheral blood, whilst transiting between SLOs. RBC are plausible candidates to deliver inhibitory signals to B cells whilst in the bloodstream, given both their abundance and decoration with a complex array of surface molecules that is rich in potential ligands [12]. Here, we test the hypothesis that contact with RBC suppresses activation of human B cells. The results reveal that RBC do inhibit B cells, including their expression of activation markers, proliferative responses and secretion of immunoglobulin, and that a key mediator of these effects is the recognition of sialic acids on the RBC surface by the B‐cell lectin CD22.

## MATERIALS AND METHODS

### Patients and donors

All donors and patients gave informed consent, and the study was approved by the North‐East of Scotland Research Ethics Committee (Application numbers: 14/NS/0009 and 13/NS/0159). Blood samples from healthy volunteers, and from patients with CLL attending Aberdeen Royal Infirmary, were collected by venepuncture into lithium heparin‐coated Vacutainers (Greiner Bio‐One). Details of patients with CLL are summarized in Table 1.

**TABLE 1:** Details of patients with CLL

Sex	Age	Binet stage	Previous therapy	Current therapy	Autoimmune status
M		C	Ibrutinib, FCR, R‐bendamustine	Corticosteroids	AIHA, previous PRCA
M	78	A	None	None	None
M	56	A	None	None	None
M	64	B	None	None	None
M	80	A	None	None	None
M	62	A	None	Corticosteroids	AIHA
F	76	B	None	Corticosteroids	AIHA
M	58	A	None	None	None
M	58	C	FCR	None	None
M	65	A	None	Corticosteroids	AIHA
M	52	A	None	None	None
F	67	C	FCR, FC, rituximab, idelalisib	None	None
M	52	A	None	None	None
F	72	A	FCR	None	None
M	69	A	None	None	None
F	76	C	None	Corticosteroids	AIHA

Abbreviations: AIHA, autoimmune haemolytic anaemia; F, female; FC, fludarabine and cyclophosphamide; FCR, fludarabine, cyclophosphamide and rituximab; M, male; PRCA, pure red cell aplasia.

### Preparation of cells

Peripheral blood mononuclear cells (PBMC) and RBC were isolated from whole blood by density gradient centrifugation on Histopaque‐1077 (Sigma‐Aldrich). The granulocyte layer was excluded when collecting RBC from the gradient, with flow cytometry confirming that the resulting preparation contains >99·99% cells staining for the RBC marker glycophorin A (CD235a). CD19^+^ B cells were purified from PBMC using the Pan B‐Cell Isolation Kit (Miltenyi Biotec), which labels other leucocytes with biotinylated antibodies to allow their depletion by Anti‐Biotin MicroBeads as per the manufacturer's instructions. Magnetic beads (Miltenyi Biotec) coated with anti‐human CD4 were used to isolate CD4^+^ T cells from PBMC as directed by the manufacturer. The purity of isolated CD19^+^ B cells and CD4^+^ T cells was confirmed by flow cytometry with, as reported in [13], >90% of selected cells CD19‐ or CD4‐positive as appropriate.

### Cell culture

Peripheral blood mononuclear cells or isolated CD19^+^ B cells were cultured at a concentration of 1·25 × 10^6^ cells/ml in the alpha modification of Eagle's medium (αMEM; Gibco), supplemented with 5% autologous serum, 4 mM l‐glutamine (Gibco), 100 U/ml sodium benzylpenicillin G (Sigma‐Aldrich), 100 μg/ml streptomycin sulphate (Sigma‐Aldrich) and 20 mM HEPES (*N*‐2‐hydroxyethylpiperazine‐*N*′‐2‐ethanesulphonic acid), pH 7·2 (Sigma‐Aldrich), in a humidified atmosphere of 5% CO_2_/95% air. PBMC or B cells were co‐cultured with RBC at ratios of 1:1, 1:10 or 1:100 for 24 h before RBC lysis (RBC Lysis Buffer; BioLegend).

### B‐cell stimulation

Peripheral blood mononuclear cells or B cells were stimulated with 5 μg/ml anti‐human CD40 (BioLegend) and 10 ng/ml interleukin (IL)‐4 (Sigma‐Aldrich) as previously described [14], prior to the addition of RBC as required.

### Flow cytometry

Flow cytometry was performed using a LSRFortessa™ (BD) or FACSDiva™(BD), with data analysed by FlowJo software v10. Zombie NIR Viability Dye (BioLegend) was used to exclude dead cells from analyses. RBC were identified by staining for glycophorin A (anti‐CD235a‐FITC, BioLegend). Lymphocytes were gated based on forward and side scatter and B cells identified as CD19^+^ (anti‐CD19‐PE, BioLegend). Malignant B cells from patients with CLL were identified as CD19^+^ and CD5^+^.

### Activation markers

Cells were washed in flow cytometry staining buffer (BD Pharmingen), treated with Fc block (BD Bioscience) and stained for flow cytometric analyses of extracellular markers using fluorochrome‐conjugated antibodies according to the manufacturer's instructions. Antibodies included anti‐CD19‐PE or anti‐CD19‐FITC, anti‐CD5‐BV421 and the activation markers anti‐HLA‐DP, DQ, DR Alexa Fluor 647 or anti‐HLA‐DP/DQ/DR‐FITC, anti‐CD86‐PerCP‐CY5.5, anti‐CD69‐FITC and anti‐CD40‐PE‐Cy7 (BioLegend); markers of maturation anti‐CD38‐FITC or anti‐CD38‐APC and CD24‐APC‐Cy7 (BioLegend); or CD22‐FITC and sialic acid‐binding immunoglobulin‐like lectin (Siglec)‐10‐APC (BioLegend). Immunoglobulin isotype‐matched control antibodies were used to confirm specificity of staining. Extracellular staining was conducted for 30 min in the dark at 4°C, before the cells were washed and analysed by flow cytometry. To correct for inter‐assay variation, ratios were calculated of staining intensity in treated versus control cultures.

### Transwell cultures

Transwell inserts (Corning) with a pore size of 0·4 µm were added to the culture plates to prevent translocation of RBC (diameter 6–8 µm). PBMC were added to the plates as described above, before addition of transwell inserts and placement of RBC into the upper chamber. After 24 h of incubation, cultures from the lower compartment were collected and analysed for changes in markers of B‐cell activation.

### Proliferation assay

After 24, 72 or 96 h of co‐culture with RBC, B cells that had been stimulated with anti‐CD40 and IL‐4 were pulsed with [^3^H]‐thymidine for 6 h. The samples were then harvested using a Tomtec harvester (Tomtec Inc.), and [^3^H]‐thymidine incorporation was measured using conventional liquid scintillation procedures (Wallac Microbeta Trilux).[15] Results were obtained as counts per minute (cpm), and stimulation indices (SI) were determined as the ratios of mean cpm in triplicate B‐cell cultures with, versus without, addition of RBC.

### Immunoglobulin ELISA

Isolated B cells were stimulated with anti‐CD40 and IL‐4, or left untreated, and cultured with or without RBC for 5 days, before the levels of IgG and IgM in culture supernatants were measured using human IgG or IgM total ELISA Ready‐SET‐Go! ™ Kit (Invitrogen).

### Antigen presentation assay

CD19^+^ B cells and CD4^+^ T cells were isolated as described above from peripheral blood of healthy donors or patients with CLL. B cells were incubated with, or without, RBC for 24 h before pulsing with either the T‐cell‐dependent primary antigen keyhole limpet haemocyanin (KLH, 10 µg/ml, Sigma‐Aldrich), the recall antigen‐purified protein derivative (PPD, 10 µg/ml, Statens Serum Institut, Copenhagen, Denmark) or the mitogen concanavalin A (ConA, 10 µg/ml, Sigma‐Aldrich) for 6 h [15]. RBC were then lysed, antigen and RBC debris were washed away, and T cells previously stained with carboxyfluorescein succinimidyl ester (CFSE) (0·2 µM, Fisher Scientific, Hampton, New Hampshire) were added at a 1:1 ratio to B cells and then cultured for 5 days. Flow cytometric determination of CFSE dilution in T cells (anti‐CD4‐PerCP‐Cy5.5) was used as a measure of their proliferation in response to B‐cell antigen presentation, with the proportions of T cells that had undergone proliferation compared in the presence versus absence of RBC.

### RBC damage

Red blood cells were resuspended in αMEM (1:20, Gibco) and were damaged by either heat shock at 56°C for 15 min, treatment with copper sulphate (CuSO4, 0·2 mM, Sigma‐Aldrich) and ascorbic acid (5 mM, Sigma‐Aldrich) for 60 min, or calcium ionophore A23187 (2 µM, Sigma‐Aldrich) for 15 min.

### Neuraminidase treatment

Sialic acid molecules were depleted from RBC by treating with neuraminidases. Both α(2,3)‐ and α(2,6)‐linked sialic acids were removed by incubating RBC in αMEM with an active recombinant fragment of *Vibrio cholerae*‐derived neuraminidase (University of St Andrews, UK) for 30 min at 37°C, before termination of the reaction by addition of 10% fetal calf serum. RBC were selectively depleted of α(2,3)‐linked sialic acids by treatment with neuraminidase S (New England Biolabs) in 1× Glycobuffer (provided) at 37ᵒC. After treatment with either enzyme, RBC were washed and resuspended in αMEM before adding to PBMC populations. RBC were analysed for sialic acid expression by flow cytometry after staining at room temperature for 30 min with the biotinylated lectins *Maackia amurensis* lectin II (MAL II, Vector Laboratories) or *Sambucus nigra* agglutinin (SNA, Vector Laboratories), which bind α(2,3)‐ or α(2,6)‐linked sialic acids respectively, followed by development with E‐Cy7‐streptavidin (eBioscience).

### Siglec blocking

To identify the activity of the Siglec family of lectins, antibodies were used to block the interaction of particular family members with their respective ligands. Polyclonal anti‐CD22 (Antibodies‐online), anti‐Siglec‐10 (Invitrogen), or the corresponding isotype controls, were added at 2·5 µg/ml to B cells prior to incubation with RBC.

### Statistical analyses

For comparison of two paired groups, non‐parametric two‐tailed Wilcoxon tests were performed. Statistical differences between ≥3 paired groups were determined using Friedman's and Dunn's tests. Statistical differences between two unpaired groups were determined using the Mann–Whitney tests. Statistical differences between ≥3 unpaired groups were determined using the Kruskal–Wallis tests. When determining statistical dependence between two variables, non‐parametric Spearman's rank tests were used. Mean +/‐ SD are shown for all data and two‐tailed *p*‐values <0·05 considered significant. Data analyses were performed using the Prism 6.0 software (GraphPad).

## RESULTS

### RBC exposure down‐regulates B‐cell activation markers

**FIGURE 1:**
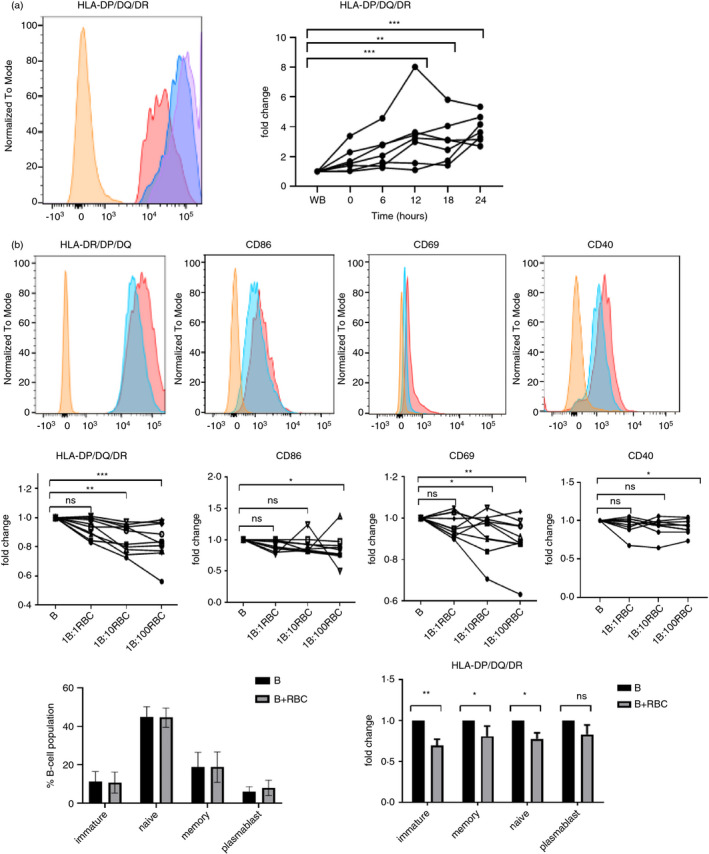
Exposure to RBC suppresses B‐cell expression of activation markers HLA‐DP/DQ/DR, CD86 and CD69. Flow cytometry was used to determine B‐cell expression of activation markers under the following conditions. (a) HLA‐DP/DQ/DR expression by B cells in whole blood versus in PBMC at intervals after separation from RBC. Representative histogram illustrates HLA‐DP/DQ/DR expression in whole blood (orange), and 6 (red), 18 (blue) and 24 (purple) hours after separation (*n* = 6). Summary graph shows fold change of HLA‐DP/DQ/DR expression by isolated B cells compared with those in whole blood. The 6 sets of linked points represent data from 6 different healthy donors. **p < 0·01, ***p > 0·001 (Friedman). (b) Isolated RBC were added back to PBMC at ratios of 1:1, 1:10 or 1:100 (B: RBC) and incubated for 24 h before determining B‐cell levels of HLA‐DP/DQ/DR, CD86, CD69 and CD40 expression. Histograms show representative data for HLA‐DP/DQ/DR, CD86, CD69 and CD40 expression in isolated B cells (red) and B cells exposed to 1:100 RBC (blue), compared with the isotype controls (orange). Summary results are expressed as fold changes compared with expression on the B‐cell‐only controls (*n* = 10). *p > 0·05, **p < 0·01, ***p > 0·001 (Friedman). (c) RBC were added to PBMC and incubated for 24 h. CD19^+^ B cells were then stained for CD24 and CD38 expression to assess maturation status. The percentages of B cells within the immature (CD19^+^CD38^hi^CD24^hi^), naïve (CD19^+^CD38^int^CD24^int^), memory (CD19^+^CD38^−^CD24^+^) and plasmablast (CD19^+^CD38^−^CD24^−^) populations are shown for B cells alone and B cells with RBC exposure (*N* = 10). (d) Expression of HLA‐DP/DQ/DR was determined for each of the maturation subsets and compared in B cells alone compared with B cells cultured with 1:100 RBC. *p< 0·5, **P < 0·01 (Friedman); B, B cells; R, 1:100 packed red blood cells; WB, whole blood

We first tested whether removal of RBC from whole blood had any effect on the expression of human B‐cell activation markers. B cells were identified as CD19^+^ and changes in their phenotype determined by flow cytometry. PBMC were separated from RBC, and B‐cell expression of HLA‐DP/DQ/DR was measured as a marker of activation at 6 hourly intervals for 24 h and compared with the expression by B cells that had remained in paired samples of whole blood. Although freshly isolated B cells already stain strongly for HLA‐DR/DP/DQ (Figure S1a), removal of RBC was associated with a notable increase in expression, which was significant by 12 h, and which typically continued to rise throughout the experiment (Figure 1a). These results indicated that B‐cell activation was inhibited by RBC at ratios found in whole blood, but for further experiments to define the extent and mechanism of suppression, ratios of 1:1 to 1:100 were tested as being more practical for cultured cells. Complementing the results of fractionating whole blood, reconstitution of PBMC with titrations of RBC resulted in corresponding, significant decreases in the levels of HLA‐DR/DP/DQ expressed by B cells (Figure 1b). In the reconstitution experiments, the levels of additional activation markers, CD86, CD69 and CD40, were measured on B cells and were also demonstrated to fall when RBC were added to PBMC (Figure 1b).

Given this evidence that RBC suppress expression of multiple B‐cell activation markers, we asked whether reconstitution of PBMC with RBC induced any changes in the maturation status of B cells. Characterization of B‐cell subsets (Figure S1b) based on CD24 and CD38 expression [16 did not show any significant changes in the percentages of immature, naïve, memory or plasmablast B cells upon addition of RBC (Figure 1c). Whether RBC suppress activation differentially between these peripheral blood subsets was determined by staining B cells for both the activation and maturation marker sets. Significant RBC suppression of B‐cell activation markers was observed in immature, naïve and memory B cells, with a trend for suppression in plasmablasts (Figure 1d).

**FIGURE 2:**
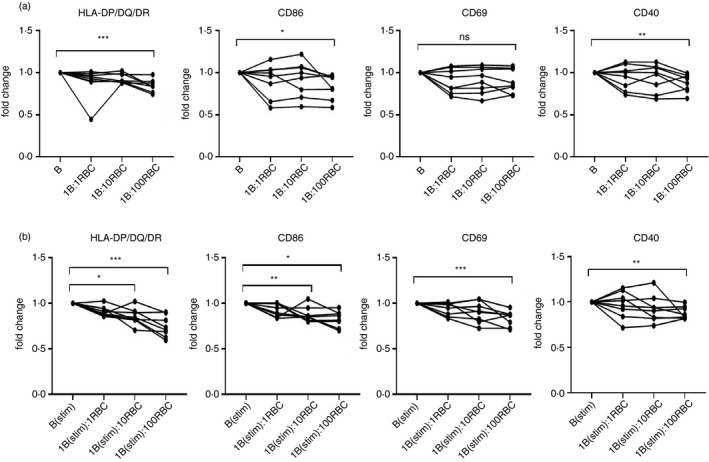
RBC suppress B cells independently of other leucocytes or stimulation status. Flow cytometry was used to determine B‐cell expression of activation markers under the following conditions. (a) CD19^+^ B cells were isolated using negative B‐cell isolation beads. RBC were added to the cultures at ratios of 1:1, 1:10 or 1:100 (B: RBC) and incubated for 24 h before determining the levels of HLA‐DP/DQ/DR, CD86, CD69 and CD40 expression on CD19^+^ B cells (*n* = 8). *p < 0·05, **p < 0·01 and ***p = 0·001 (Friedman). (b) B cells stimulated with 5 µg/ml anti‐human CD40 and 10 ng/ml IL‐4 were co‐incubated with 1:1, 1:10 or 1:100 (B: RBC) and incubated for 24 h before determining the levels of HLA‐DP/DQ/DR, CD86, CD69 and CD40 expression on CD19^+^ B cells (*n* = 8). *p < 0·05, **p < 0·01 and ***p < 0·001 (Friedman). Results are shown as fold change compared with the B‐cell‐only culture. B, B cells; RBC, red blood cells; stim, stimulated

We also determined whether the presence of other leucocytes in PBMC was necessary for the suppressive effect of RBC on B‐cell activation markers. When purified populations of CD19^+^ B cells were isolated from PBMC, their expression of HLA‐DP/DQ/DR, CD86 and CD40 again fell significantly when cultured together with RBC (Figure 2a), indicating that the inhibitory interaction between RBC and B cells was independent of any other cell type. Another question addressed was whether the suppression of activation markers by RBC was also seen when B cells were responding to stimulation, and not an effect limited to resting cells. When purified B cells were stimulated with anti‐CD40 antibody and IL‐4, the expression of HLA‐DR/DP/DQ, CD86, CD69 and CD40 rose as expected, but the increases were significantly lower in co‐cultures containing RBC (Figure 2b). Overall, these results demonstrate that the presence of RBC regulates the activation level of B cells, both resting and stimulated, and independently from their differentiation status, and that this suppressive effect does not require the presence of other leucocyte types.

### RBC exposure inhibits B‐cell function

**FIGURE 3:**
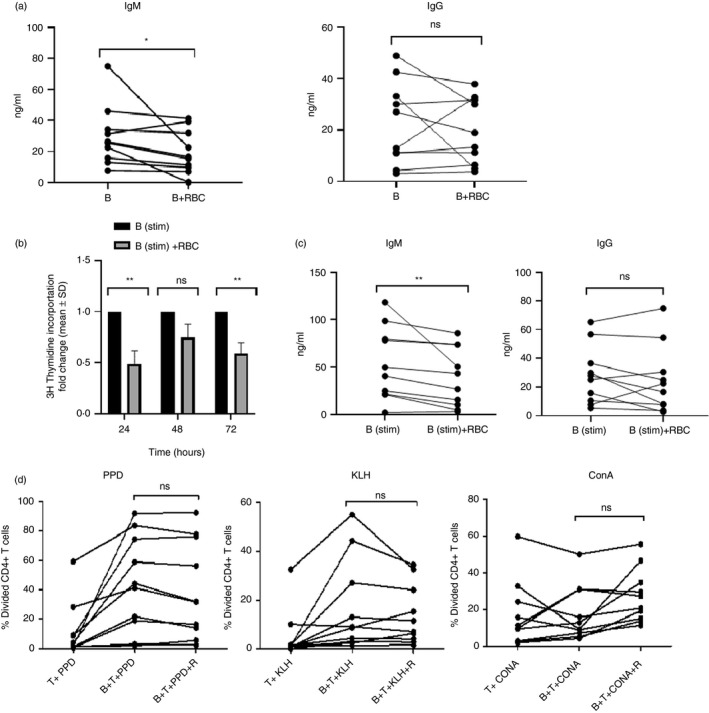
Exposure to RBC suppresses B‐cell IgM secretion and proliferation, and can reduce ability to present antigen. (a) B cells without or with RBC (1:100) were incubated for 5 days before levels of IgM and IgG were measured in supernatants by ELISA (*n* = 10). **p < 0·01 (Wilcoxon). (b) B cells were stimulated with anti‐CD40 and IL‐4, and RBC added to cultures (1B:100RBC) and incubated for 24, 48 or 72 h prior to ^3^H‐thymidine incorporation. Proliferation results are presented as fold changes of mean counts per minute in B‐cell–RBC co‐cultures compared with B‐cell‐only cultures (*n* = 8). **p < 0·01 (Wilcoxon). (c) CD19^+^ B cells were isolated and stimulated with 5 µg/ml anti‐CD40 and 10 ng/ml IL‐4 before addition of RBC (1B:100RBC). Levels of IgM and IgG were measured in supernatants by ELISA after 5 days of culture. **p < 0·01 (Wilcoxon). (d) Flow cytometric determination of the percentage of CFSE‐labelled CD4^+^ T cells that had divided was used as a measure of B‐cell antigen presentation in cultures of T cells exposed to B cells that had been pulsed with primary antigen PPD or secondary antigen KLH, or stimulated non‐specifically with ConA in the presence or absence of 1:100 RBC (*n* = 10) (Wilcoxon). B, B cells; ConA, concanavalin A; Ig, immunoglobulin; KLH, keyhole limpet haemocyanin; ng/ml, nanograms per millilitre; PPD, purified protein derivative; RBC, red blood cells; SD, standard deviation

The next question to be addressed was whether exposure to RBC affects B‐cell function; in particular proliferation, release of immunoglobulin and the ability to present antigen to T cells. When purified, unstimulated CD19^+^ B cells were incubated in the presence of RBC, there were modest but significant decreases in the levels of IgM, but not IgG, secreted into the supernatant (Figure 3a). Although B cells are relatively short‐lived in culture, the levels of immunoglobulin are cumulative and therefore represent their initial secretory activity. Co‐incubation with RBC also inhibited proliferative (Figure 3b) and IgM secretory (Figure 3c) responses of B cells to anti‐CD40 antibody and IL‐4, demonstrating that the suppressive effects of RBC on function were, like those on activation marker expression, seen in both stimulated and unstimulated B cells. The flow cytometric analyses of changes in activation (Figure 1b) and maturation (Figure 1b) marker and expression are consistent with RBC suppressing the B‐cell fraction as a whole rather than particular subpopulations. Although the biological relevance of any of the changes in B‐cell function *in vitro* remains to established, taken together, these data point to RBC as regulators of a wide range of B‐cell activities.

The ability of RBC to suppress B‐cell expression of HLA‐DP/DQ/DR and CD86, which are two key components of the antigen‐presenting machinery, prompted us to test whether RBC inhibited antigen‐presenting function. The proliferation of purified, CFSE‐stained CD4^+^ T cells presented with antigen by purified CD19^+^ B cells was compared after the B cells had been incubated in the presence or absence of RBC. The proportions of CD4^+^ T cells proliferating in response to the antigens PPD or KLH were reduced when the presenting B cells had been preincubated with RBC for cells from seven of the ten donors tested, although the fall did not achieve statistical significance across the donor group overall (Figure 3d). By contrast, suppressive effects of RBC preincubation with B cells on T‐cell responses were infrequently observed when cultures were stimulated with the mitogen concanavalin A, which does not require antigen processing and presentation as peptide on MHC class II. Thus, RBC suppress a variety of B‐cell functions, including proliferation and IgM secretion, and can inhibit the ability to present MHC‐dependent antigens in many, but not all, donors.

### RBC‐mediated B‐cell suppression is contact and sialic acid‐dependent

**FIGURE 4:**
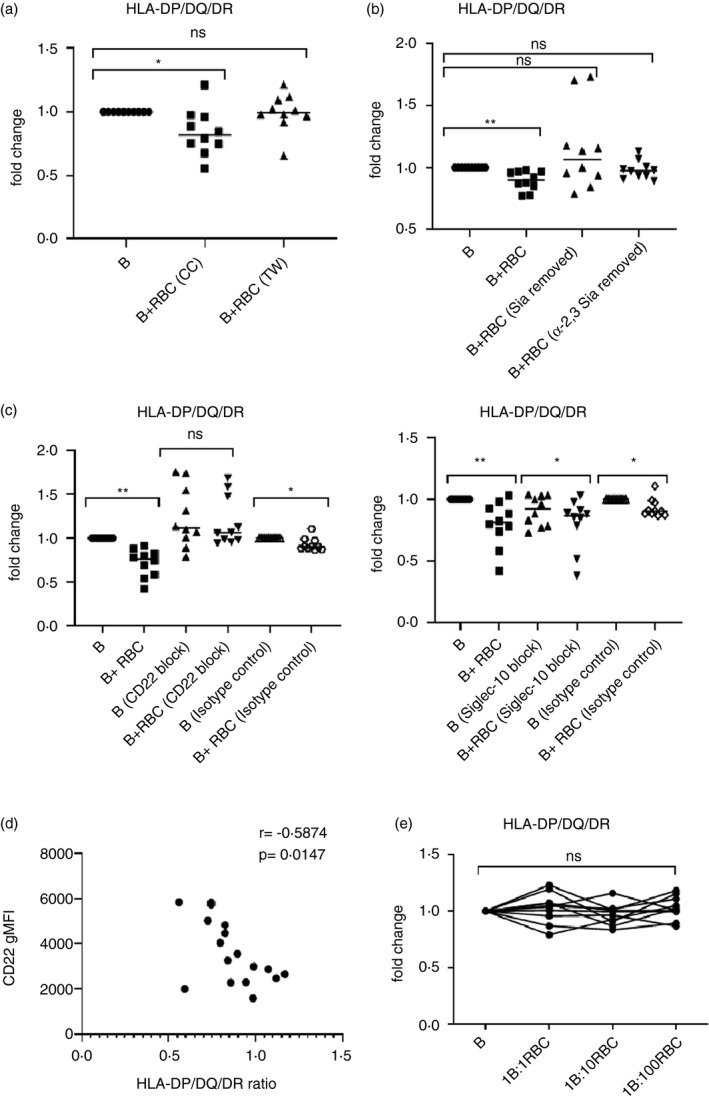
RBC‐induced B‐cell suppression is contact, sialic acid and Siglec‐dependent. Flow cytometry was used to determine B‐cell expression of activation markers under the following conditions. (a) After 24 h of incubation, HLA‐DP/DQ/DR expression on CD19^+^ B cells was compared in B cells cultured alone, co‐cultured with RBC, or in cultures with B cells separated from RBC by transwell inserts. Results expressed as fold changes compared with B‐cell‐only cultures (*n* = 10). *p < 0·05 (Friedman). (b) HLA‐DP/DQ/DR expression on CD19^+^ B cells was compared after 24 h of culture alone, or co‐incubated with RBC that had been treated with neuraminidase or neuraminidase S respectively to remove both α(2,3)‐ and α(2,6)‐linked, or only α(2,3)‐linked, sialic acids. Results expressed as fold changes compared with B‐cell‐only cultures (*n* = 10). **p < 0·01 (Kruskal–Wallis). (c) HLA‐DP/DQ/DR expression was compared on CD19^+^ B cells that had been treated with blocking anti‐CD22 or anti‐Siglec‐10 antibodies, or an isotype control, before 24 h of culture alone, or co‐incubated with RBC. Results expressed as fold changes compared with untreated B‐cell‐only control cultures (*n* = 10). *p < 0·05, **p < 0·01 (Wilcoxon). (d) CD19^+^ B cells were analysed for expression of CD22, and this was correlated with the fold change in HLA‐DP/DQ/DR expression after RBC co‐culture in individual donors (*n* = 17). Spearman's *r* = −0·5874, *p < 0·05. (e) CD19^+^ B cells were isolated from CLL patients, autologous RBC were added to the cultures at ratios of 1:1, 1:10 or 1:100 (B: RBC) and incubated for 24 h before determining the levels of HLA‐DP/DQ/DR expression on CD19^+^CD5^+^ B cells (*n* = 10) (Friedman). B, B cells; CC, co‐culture; gMFI, geometric mean fluorescence intensity; RBC, 1:100 red blood cells; Sia, sialic acids; TW, transwell

The next set of experiments characterized the mechanisms responsible for RBC regulation of B cells. We first established whether the suppression was due to direct cell–cell interactions, or the release of soluble factors, by culturing purified CD19^+^ B cells and RBC in the separate chambers of a transwell system. When physical contact between co‐cultured B cells and RBC was prevented in this way, the effect of RBC in reducing the expression of the activation markers HLA‐DP/DQ/DR was lost (Figure 4a).

The dependence of RBC suppression on direct contact with B cells raises the question as to the identities of the ligand–receptor interactions responsible. Suppression was limited to healthy RBC, as RBC modified by oxidation or other forms of damage failed to suppress B‐cell activation (Figure S2). The glycocalyx of RBC is particularly prominent and rich in sialic acids [17], which can be lost when RBC membranes have been damaged [18, 19], so we tested whether the inhibitory effect of RBC on purified CD19^+^ B cells is dependent on these glycans. RBC pretreated with a recombinant *Vibrio cholerae* neuraminidase fragment containing the active site that cleaves both α(2‐3)‐ and α(2‐6)‐linked terminal sialic acids from the membrane (Figure S3a) [20] lost the ability to down‐regulate B‐cell expression of HLA‐DP/DQ/DR (Figure 4b). RBC pretreated with the recombinant enzyme neuraminidase S, which cleaves α(2‐3)‐ but not α(2‐6)‐linked terminal sialic acids (Figure S3b) down‐regulated B‐cell expression of HLA‐DP/DQ/DR marginally, an effect that was no longer statistically significant (Figure 4b). Thus, these initial mechanistic studies showed that the inhibitory effects of RBC on B cells are dependent on direct cell–cell contact between the populations, and on the display of sialic acids by RBC, with both α(2‐6)‐ and α (2‐3)‐linked sialic acids required for optimal suppression.

### RBC‐mediated B‐cell suppression is dependent on B‐cell CD22, but not Siglec‐10

The dependence of RBC suppression on sialic acids suggested the identities of the B‐cell receptors involved. CD22 and Siglec‐10 are inhibitory co‐receptors on B cells [21, 22], and each acts as a lectin to bind the terminal sialic acid residues on cell surface glycans. We therefore tested whether the inhibitory effect of RBC on purified CD19^+^ B cells is dependent on CD22 or Siglec‐10 recognition of these sugars. The ability of RBC to down‐regulate B‐cell HLA‐DP/DQ/DR was prevented by the addition of a blocking antibody specific for CD22 (Figure 4c), but not an antibody for Siglec‐10 (Figure 4c), to co‐cultures. In some individuals, blocking CD22 on purified B cells in the absence of RBC also increased expression of HLA‐DR/DP/DQ, but no direct stimulatory effects of anti‐CD22 antibodies have been recorded, and the result is instead consistent with blockade of tonic inhibitory signals from CD22 ligation *in cis* that have been reported to arise because the receptor itself is glycosylated and decorated with sialic acids [23].

If CD22 mediates suppression of B cells by RBC, and given the observation that expression of this receptor varies markedly between donors (Figure S4a), it would be predicted that its levels determine the strength of the inhibitory effect. This prediction was fulfilled as there is a significant positive correlation between CD22 expression on B cells and the reduction in HLA‐DP/DQ/DR after incubation with RBC (Figure 4d). To further test the role of CD22, and to explore the relevance of the RBC suppressive mechanism to a human disease, we took advantage of findings that the lectin CD22 is expressed at only low levels by malignant B cells in patients with CLL [24]. We hypothesized that that these low lectin levels would result in refractoriness to RBC inhibition and so might contribute towards the activated phenotype [9, 25] of CLL cells. Analyses of peripheral blood samples from cases of CLL confirmed that CD19^+^ B cells with the malignant CD5^+^ phenotype, which predominate in patients’ PBMC, had low levels of CD22, and their expression of Siglec‐10 was also found to be somewhat reduced (Figure S4b). In contrast to the inhibitory effects of RBC on CD19^+^ B cells from healthy donors, which were replicated for the corresponding minor CD5^+^ CD19^+^ B‐cell fractions (Figure S5a), the addition of RBC failed to reduce the expression of HLA‐DP/DQ/DR (Figure 4e) by CD5^+^ CD19^+^ B cells from patients with CLL.

We also asked whether the ability of CLL cells to present autoantigen to T cells might resist inhibition by RBC, as CLL is closely associated with autoimmune haemolytic anaemia (AIHA), and the malignant B cells are effective APC in driving the pathogenic responses, which most commonly target the RhD protein [15]. CD5^+^ CD19^+^ CLL B cells were incubated with or without RBC, pulsed with RhD protein, and cultured with autologous CD4^+^ T cells that had been CFSE‐stained to follow their proliferation. In six of the 11 patients tested, exposure of CLL B cells to RBC failed to reduce the T‐cell proliferative response, but RBC were suppressive in the remaining five cases, and lack of inhibition was not related to whether the patients also exhibited clinical AIHA (Figure S5b).

Taken together, these results establish ligation of RBC sialic acid by the B‐cell lectin receptor CD22 as a key molecular mechanism in the suppression of B cells by RBC, and illustrate that the effectiveness of this mechanism is reduced in a human disease characterized by B cells with an activated phenotype.

## DISCUSSION

Our work has revealed a novel function of RBC, the suppression of B cell activation. RBC were shown to inhibit B‐cell display of activation markers (HLA‐DR/DP/DQ, CD40, CD69, CD86), cellular proliferation in response to CD40 and IL‐4, and IgM secretion. Our results contribute to current models of crosstalk between the immune system with other cells and demonstrate that RBC functions extend beyond gaseous transport to include regulation of immune responsiveness.

RBC‐induced suppression of B cells was found to be contact‐dependent, with a major molecular mechanism identified as ligation of sialic acid on the RBC surface by the inhibitory B‐cell receptor CD22. Both α(2‐3)‐ and α(2‐6)‐linked terminal sialic acids are abundant as prospective ligands on RBC [26], but CD22 recognizes only the latter [27]. CD22 ligation with α(2‐6)‐linked sialic acids recruits the tyrosine phosphatase, Src homology 2 domain‐containing phosphatase 1, to immunoreceptor tyrosine‐based inhibitory motifs and so inhibits B‐cell receptor‐induced calcium signalling [21], and also inhibits stimulatory signals from other innate receptors [28]. The ability of CD22 to bind sialic acid has previously been recognized as important in the induction of B‐cell tolerance to self‐antigens, with sialylated glycans being characteristic of higher vertebrates but absent on microbes [29]. The role of trans interactions of CD22 with sialic acids in tolerance has been demonstrated using sialylated multivalent antigens that were able to bind both the BCR and CD22. Exposure to such antigens resulted in the dampening of BCR signalling [30]. We now infer that ligation of B‐cell CD22 by sialic acids on other cell types, such as RBC, can also play a role in regulating B‐cell activity. This complements the work of Spiller *et al*, who showed that the display of hen egg lysozyme (HEL) antigen by C57BL/6J mouse RBC, which naturally lack CD22 and Siglec‐10 ligands, resulted in strong activation of HEL‐specific B cells, but that these responses were lost if synthetic high‐affinity ligands had been introduced to the RBC membrane [31]. These animal studies support the mechanism of B‐cell inhibition that we propose, but also reveal species differences as, unlike the situation reported in mice, we show that sialic acids naturally displayed by human RBC act as effective ligands for inhibitory lectin receptors on B cells. CD22^−/−^ gene‐deleted mice have been widely used to determine the role of CD22 as an inhibitory co‐receptor on B cells [32]. Further comparison of the detailed phenotype of such mice with our work is problematic because they can also carry other unintended genetic changes that result in, for example, an intrinsic RBC defect [33].

Although CD22 was determined to be a key B‐cell inhibitory receptor ligated by sialic acids on human RBC, the suppressive effect was no longer significant after RBC were selectively depleted of α(2‐3)‐sialic acids, which are not recognized by CD22 [27]. This raised the possibility of a contribution via another receptor, Siglec‐10, the candidate B‐cell lectin that does bind α(2‐3)‐sialic acids [34], with previous studies demonstrating that cells transfected to express this lectin gained the ability to bind human RBC in a sialic acid‐dependent manner [35]. However, any contribution of Siglec‐10 to the suppressive mechanism we describe appears minor, as RBC retain a significant inhibitory effect on B cells after blockade of this receptor.

We propose that RBC suppress human B cells in order to prevent their activation in an inappropriate environment, the bloodstream. This effect is dependent on continued physical contact and therefore would be lost to allow the full responsiveness of B cells when they migrate into tissues such as SLO, which provide the cellular architecture necessary for the activation and control of adaptive immune responses [36]. Sialic acids are widely expressed on human cellular surfaces, but the density and diversity of sialylated glycans on RBC [17] may equip them to be particularly effective immune regulators in the bloodstream.

Activation of adaptive immune cells such as B cells outside SLO in the bloodstream may not only be inefficient in generating protective immunity, but could compromise control of unwanted or damaging B‐cell function in autoimmune or neoplastic disease. There is evidence that ineffective CD22 and Siglec‐10 ligation contribute to disease. For example, CD22 polymorphisms are reported to predispose to autoimmunity, with the low‐expressing allele CD22^a^ found in strains such as the lupus‐prone BXSB mice [37]. Furthermore, an autoimmune phenotype involving high levels of autoantibody and mild glomerulonephritis was found in ageing C57BL/6 mice with Siglec‐10 deficiency [38]. Here, we demonstrate ineffective interactions between RBC and CLL B cells, which express low levels of CD22. CLL B cells were found to be refractory to the down‐regulatory effects of RBC on activation, which may be a previously unrecognized factor contributing to the activated phenotype of CLL B cells.

Whether RBC inhibit antigen presentation by healthy B cells, in addition to suppressing other signs of activation, differed between donors. Furthermore, although effects in individual patients cannot be ruled out, there was no clear evidence that failure of RBC to regulate CLL B cells contributes to their ability [15] to present autoantigen and so promote autoimmune complications such as AIHA in CLL. In several patients, RBC did not block CLL B‐cell presentation of RhD protein, the major autoantigen in AIHA, but, as above, comparable results were also obtained with foreign antigen and healthy B cells in some donors, and there also was no correlation with the diagnosis of concurrent AIHA. However, subclinical autoimmunity is common in CLL [11] and may confound this analysis. Low expression of CD22 may be compensated for by other mechanisms for regulating B‐cell activation in particular individuals. For instance, Giovannone et al recently demonstrated a role for the immunomodulatory lectin galectin‐9 (Gal‐9) in BCR signalling and regulation by ligating CD45 on naïve B cells [39].

RBC have been reported to exert modulatory effects on neutrophils [5], monocytes [40] and T cells [6]. Neutrophil activation was shown to be dampened by RBC sialic acids interacting with the inhibitory receptor Siglec‐9 [5], and RBC induced immunosuppressive effects on healthy monocytes in an *in vitro* model of RBC transfusion [40]. A suppressive effect on lymphocytes has been shown with RBC significantly suppressing mitogen‐stimulated human T‐cell proliferation in a dose‐dependent manner [6], and a suppressive effect on antigen‐presenting cells has also been observed: a subset of dendritic cells (DC) known as slanDC are early‐producers of IL‐12 and are found at high frequencies in type 1T helper cell‐associated inflammatory diseases. Ligation of signal‐ regulatory protein α on slanDC by CD47 on RBC in the bloodstream inhibited the maturation and pro‐inflammatory effects of these cells [41].

Overall, this work helps to develop the paradigm that RBC regulate immune cells. Our demonstration that RBC suppress a major arm of the adaptive immune system, the B‐cell compartment, suggests a model whereby RBC are important general regulators of immune cells whilst they are circulating in the bloodstream.

## Conflict of interest

None.

## Author contributions

CSL designed and performed experiments and wrote the paper; HC designed and performed experiments; AMH designed experiments and supervised the work; MAV supervised the work and wrote the paper; RNB supervised the work and wrote the paper.

## Supporting information

Fig S1‐S5

## Data Availability

Data are available on reasonable request from the corresponding author.
