# Supplementary material for: The red blood cell as a novel regulator of human B‐cell activation
Source: Immunology. 2021 May 6;163(4):436–47. doi: 10.1111/imm.13327 (PMC8274151; doi:10.1111/imm.13327)
Supplement: Supplementary file 1 — Fig S1‐S5 [file IMM-163-436-s001.pptx]

## Slide 1
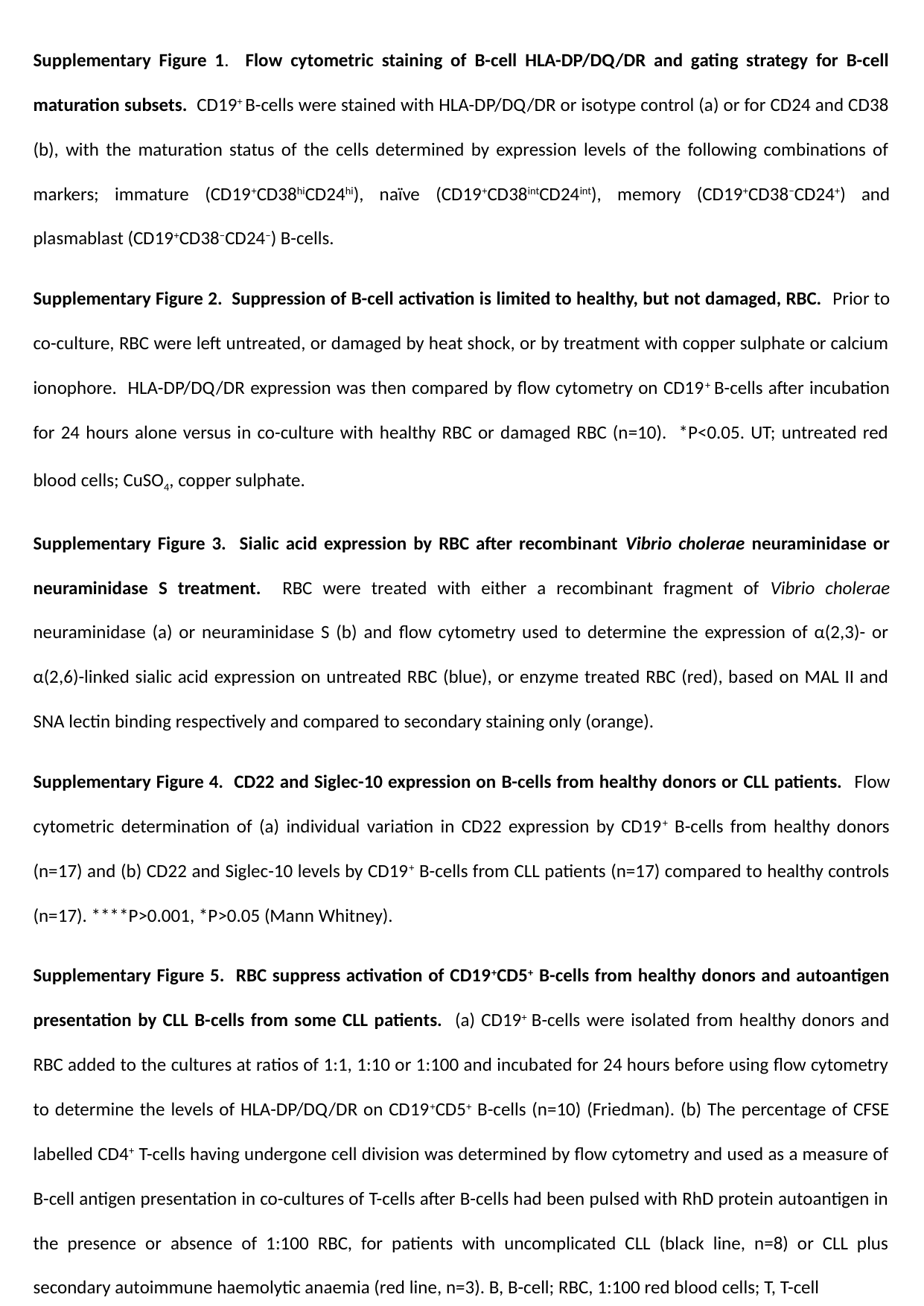

Supplementary Figure 1. Flow cytometric staining of B-cell HLA-DP/DQ/DR and gating strategy for B-cell maturation subsets. CD19+ B-cells were stained with HLA-DP/DQ/DR or isotype control (a) or for CD24 and CD38 (b), with the maturation status of the cells determined by expression levels of the following combinations of markers; immature (CD19+CD38hiCD24hi), naïve (CD19+CD38intCD24int), memory (CD19+CD38−CD24+) and plasmablast (CD19+CD38−CD24−) B-cells.
Supplementary Figure 2. Suppression of B-cell activation is limited to healthy, but not damaged, RBC. Prior to co-culture, RBC were left untreated, or damaged by heat shock, or by treatment with copper sulphate or calcium ionophore. HLA-DP/DQ/DR expression was then compared by flow cytometry on CD19+ B-cells after incubation for 24 hours alone versus in co-culture with healthy RBC or damaged RBC (n=10). *P<0.05. UT; untreated red blood cells; CuSO4, copper sulphate.
Supplementary Figure 3. Sialic acid expression by RBC after recombinant Vibrio cholerae neuraminidase or neuraminidase S treatment. RBC were treated with either a recombinant fragment of Vibrio cholerae neuraminidase (a) or neuraminidase S (b) and flow cytometry used to determine the expression of α(2,3)- or α(2,6)-linked sialic acid expression on untreated RBC (blue), or enzyme treated RBC (red), based on MAL II and SNA lectin binding respectively and compared to secondary staining only (orange).
Supplementary Figure 4. CD22 and Siglec-10 expression on B-cells from healthy donors or CLL patients. Flow cytometric determination of (a) individual variation in CD22 expression by CD19+ B-cells from healthy donors (n=17) and (b) CD22 and Siglec-10 levels by CD19+ B-cells from CLL patients (n=17) compared to healthy controls (n=17). ****P>0.001, *P>0.05 (Mann Whitney).
Supplementary Figure 5. RBC suppress activation of CD19+CD5+ B-cells from healthy donors and autoantigen presentation by CLL B-cells from some CLL patients. (a) CD19+ B-cells were isolated from healthy donors and RBC added to the cultures at ratios of 1:1, 1:10 or 1:100 and incubated for 24 hours before using flow cytometry to determine the levels of HLA-DP/DQ/DR on CD19+CD5+ B-cells (n=10) (Friedman). (b) The percentage of CFSE labelled CD4+ T-cells having undergone cell division was determined by flow cytometry and used as a measure of B-cell antigen presentation in co-cultures of T-cells after B-cells had been pulsed with RhD protein autoantigen in the presence or absence of 1:100 RBC, for patients with uncomplicated CLL (black line, n=8) or CLL plus secondary autoimmune haemolytic anaemia (red line, n=3). B, B-cell; RBC, 1:100 red blood cells; T, T-cell

## Slide 2
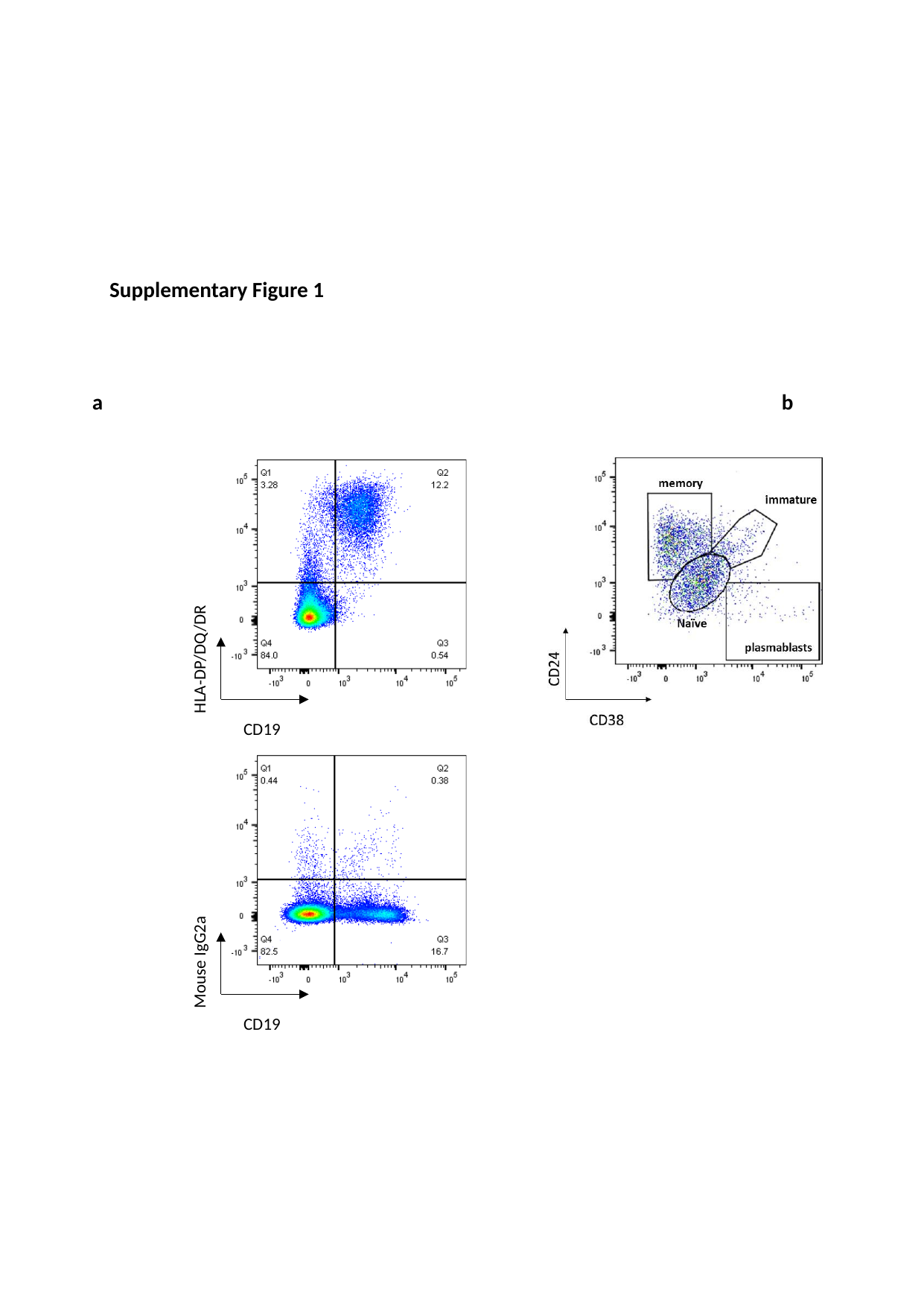

Supplementary Figure 1
 a						 b
HLA-DP/DQ/DR
CD19
Mouse IgG2a
CD19

## Slide 3
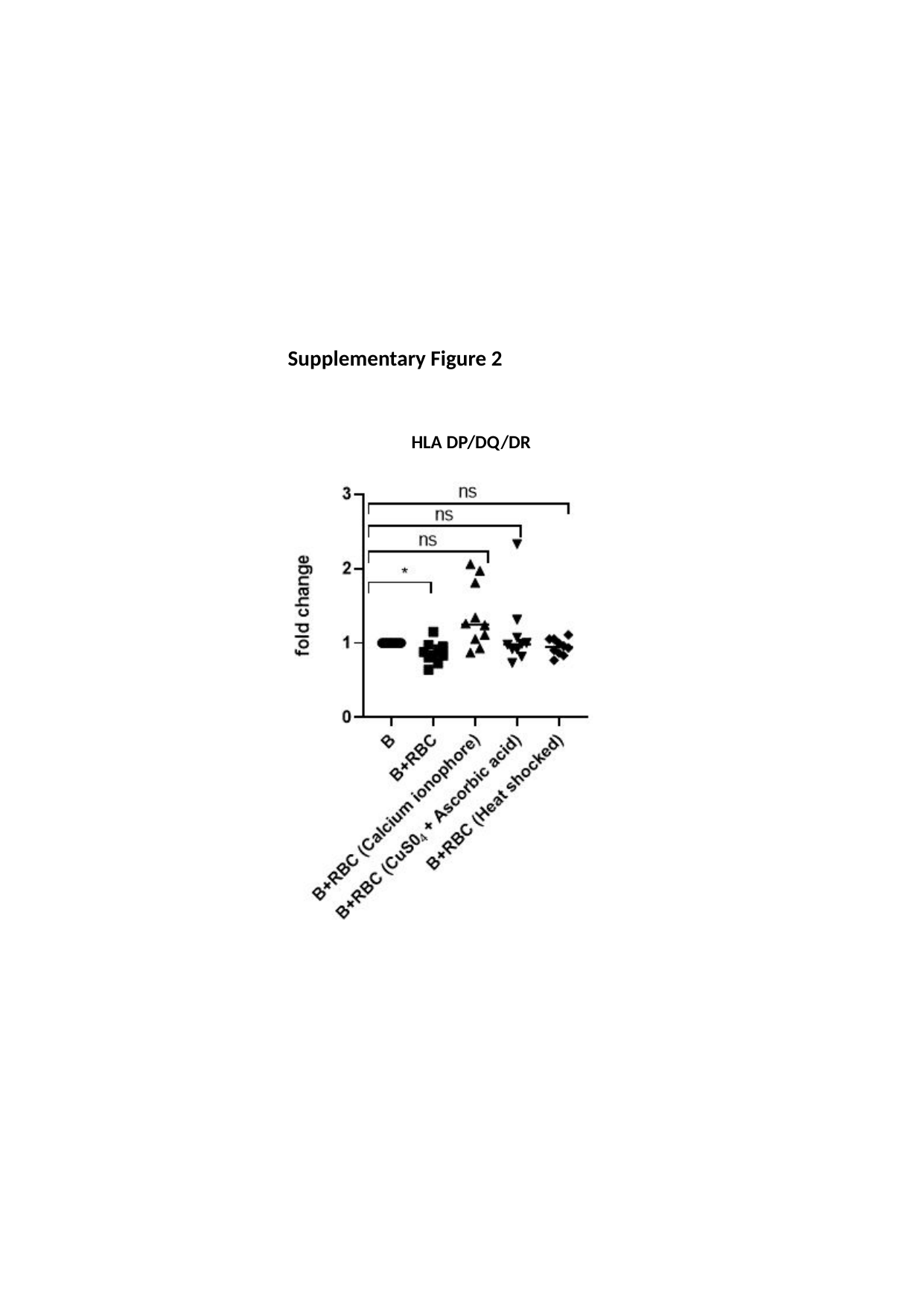

Supplementary Figure 2
HLA DP/DQ/DR

## Slide 4
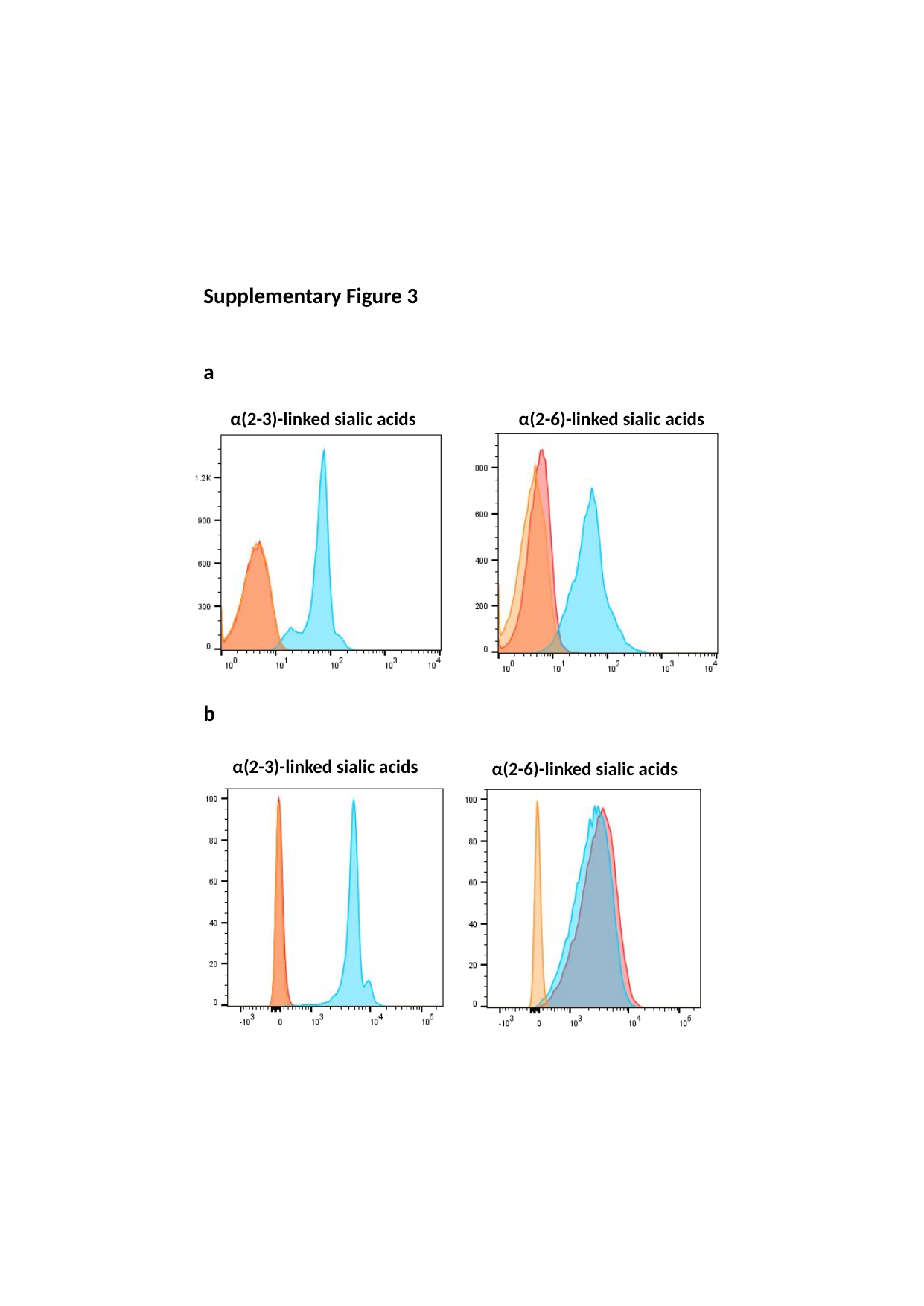

Supplementary Figure 3
a
α(2-3)-linked sialic acids
α(2-6)-linked sialic acids
b
α(2-3)-linked sialic acids
α(2-6)-linked sialic acids

## Slide 5
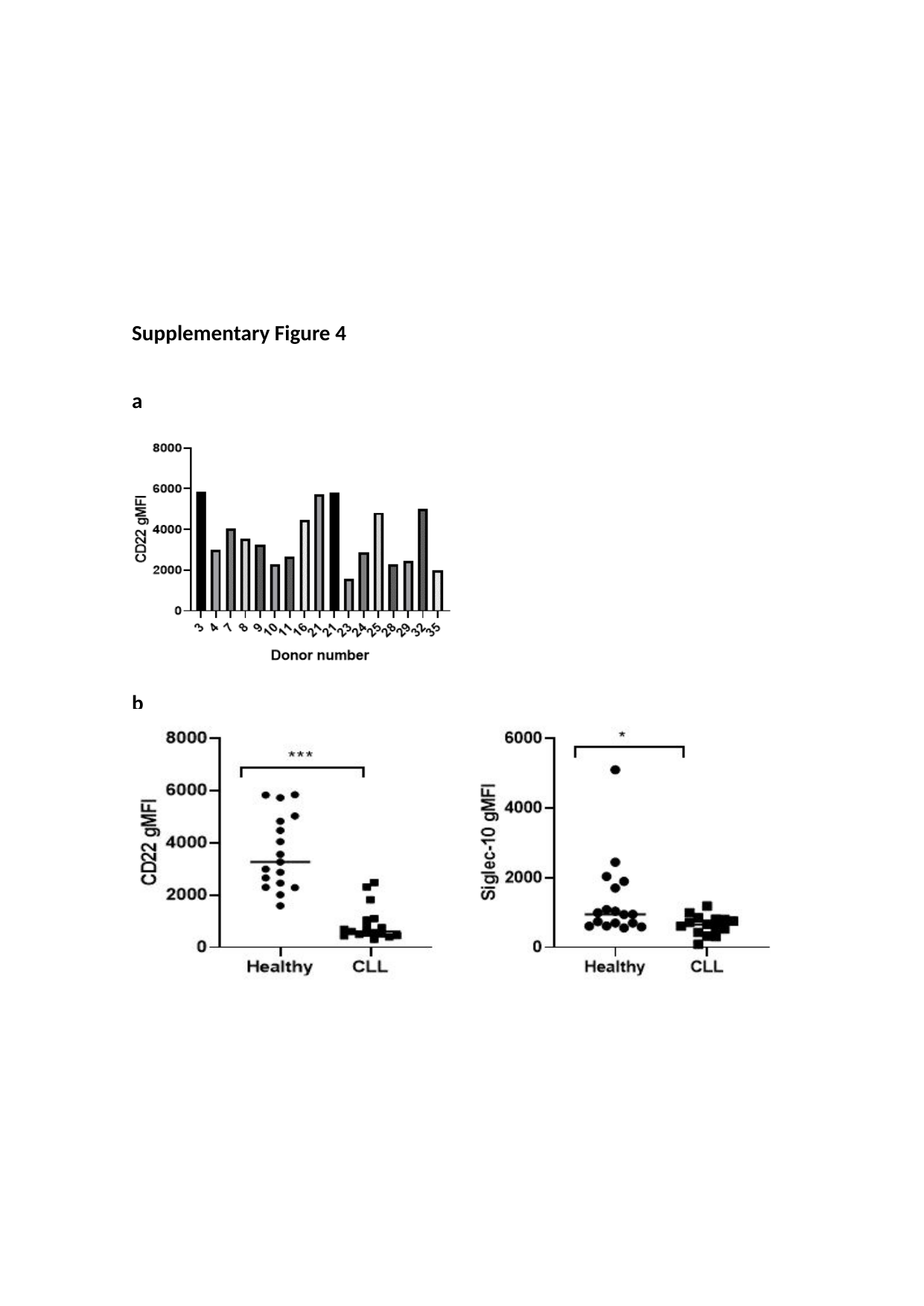

Supplementary Figure 4
a
b

## Slide 6
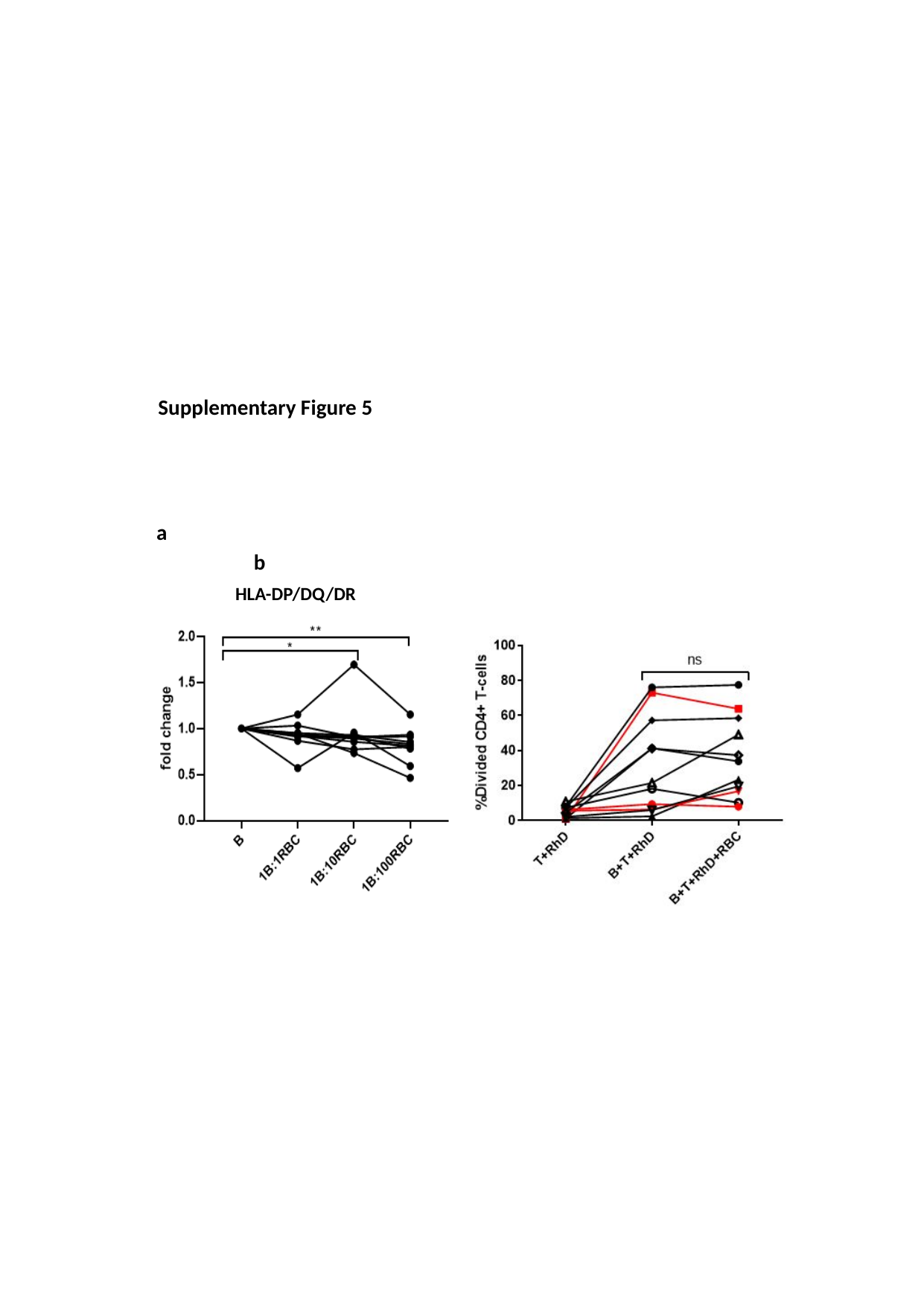

Supplementary Figure 5
 a						b
HLA-DP/DQ/DR
